# Circular RNA expression profiling reveals that circ-PLXNA1 functions in duck adipocyte differentiation

**DOI:** 10.1371/journal.pone.0236069

**Published:** 2020-07-21

**Authors:** Laidi Wang, Wenshuang Liang, Shasha Wang, Zhixiu Wang, Hao Bai, Yong Jiang, Yulin Bi, Guohong Chen, Guobin Chang

**Affiliations:** 1 Key Laboratory of Animal Genetics and Breeding and Molecular Design of Jiangsu Province, Yangzhou University, Yangzhou, China; 2 Joint International Research Laboratory of Agriculture and Agri-Product Safety, the Ministry of Education of China, Institutes of Agricultural Science and Technology Development, Yangzhou University, Yangzhou, China; East Tennessee State University, UNITED STATES

## Abstract

Adipocytes are derived from pluripotent mesenchymal stem cells through adipogenesis. Pre-adipocyte differentiation in poultry greatly influences fat deposition and meat quality. Circular RNAs (circRNAs) have an important function in cancer and some differentiation processes. Herein, high-throughput transcriptome sequencing was used to detect circRNAs present in cherry valley duck pre-adipocyte and adipocyte differentiation over 3 days. We identified 9,311 circRNAs and 141 differentially expressed circRNAs. Sequencing results were verified through qRT-PCR using seven randomly selected circRNAs, and competing endogenous RNA (ceRNA) networks were exhibited by ten important circRNAs in duck adipocyte differentiation. circRNA plexin A1 (circ-PLXNA1) was detected in duck adipocytes and mainly expressed in adipose, leg muscle and liver. Inhibition of circ-PLXNA1 limited the differentiation of duck adipocyte. There were four corresponding miRNAs for circ-PLXNA1 and 313 target genes for those miRNAs. CeRNA“circ-PLXNA1/miR-214/CTNNB1 axis” was focused and verified by a dual-luciferase reporter experiment. After co-transfection of cells with si-circ-PLXNA1 and miR-214 mimics, the expression level of CTNNB1 was down-regulated, triglyceride content and the adipogenic capacity of preadipocytes decreased. While there were no significant change after si-CTNNB1 transfection. All these results provide further insight into the circRNAs, especially for circ-PLXNA1 in duck adipocyte differentiation.

## Introduction

The intramuscular fat content of livestock has received much attention due to its high contribution to meat quality. Understanding or manipulating the process may, therefore, lead to better meat quality. Fat deposition is influenced by both the number of fat cells and the enlargement of adipocytes in muscle. The proliferation and differentiation of pre-adipocytes lead to the formation of adipose tissue, and pre-adipocyte differentiation is characterized by a coordinated increase in gene expression and lipid droplet accumulation [1–3]. In poultry, due to the low capacity of adipose tissue to perform *de novo* lipogenesis [4], dexamethasone, insulin, 3-isobutyl-1-methylxanthine (IBMX), and especially oleic acid are supplemented into culture medium to induce pre-adipocyte differentiation *in vitro*. Lipoprotein-lipase (LPL), CCAAT/enhancer-binding protein-α (C/EBPα), peroxisome proliferator-activated receptor γ(PPARγ), fatty acid synthetase (FAS), and adipocyte fatty-acid binding protein 4 (FABP4) are usually highly expressed as marker genes during pre-adipocyte differentiation [5]. Delta-like non-canonical Notch ligand 1 (DLK1) is a molecular gatekeeper of adipogenesis that acts by maintaining the pre-adipocyte state and preventing adipocyte differentiation [6]. Pre-adipocyte differentiation is a complex biological process that has been extensively studied using a variety of mammal cell lines. Currently, the differentiation mechanism for duck adipocytes differentiation is not clear.

CircRNAs are a novel class of endogenous non-coding RNAs characterized by a stable and covalently closed loop [7,8]. These circRNAs are divided into subtypes, including cytoplasmic circRNAs, nuclear circRNAs, and exon-intron circRNAs (EIciRNAs) by diversifications in sequence composition and cellular localization [9,10]. Functional studies show that circRNAs participate in various physiological and pathological processes, including cell survival, apoptosis, differentiation, and metastasis [11–13]. CircRNAs can also function as competing endogenous RNAs (ceRNAs) [14,15]. Additionally, circRNA homeodomain-interacting protein kinase 3 (circ-HIPK3) promotes the proliferation and differentiation of chicken myoblast cells through competitive adsorption of miR-30a-3p [11] and many circRNAs are involved in vascular smooth muscle cell function [16].

CircRNAs have been charachterized in liver tissue and myoblast in chicken [17,18]. We generated RNA sequencing data from duck pre-adipocytes and those that had been differentiating for 3 days and identified 9,311 circRNA candidates. In total, 141 differentially expressed (DE)-circRNAs were involved in duck adipocyte differentiation. We further analysed one circRNA produced by the PLXNA1 gene, circ-PLXNA1. In ducks, the PLXNA1 gene was located at chromosome 13 and the expression level of circ-PLXNA1 was positively correlated with the differentiation progress. Our result verified the target function of circ-PLXNA1/miR-214 and miR-214/CTNNB1 in vitro, and based on this information we tried to explore the function of circ-PLXNA1 in duck pre-adipocyte differentiation.

## Materials and methods

### Ethics statement

All animal experimental procedures were approved and guided by the Institutional Animal Care and Use Committee of Yangzhou University (approval number: 176–2019).

### Duck pre-adipocyte isolation and culture

Primary pre-adipocytes were isolated from the 20-day-old embryonic subcutaneous adipose tissue of cherry valley ducks, using the method established previously in our prophase research (Wang et al., 2018a). Briefly, embryos were rapidly surgically removed out and subcutaneous adipose tissue was dissected from the leg. After washing in PBS, the adipose tissue was minced into 1 mm^3^ sections and incubated in 0.1% collagenase type I (Gibco, USA) for 60 min at 37°C. Growth medium composed of Dulbecco's Modified Eagle's medium (DMEM) (Hyclone, USA), 10% fetal bovine serum (FBS) (Gibco, USA), 100 U/mL penicillin (Solarbio, China), and 100 μg/mL streptomycin (Solarbio, China) were used to end the digestion. The mixture was filtered through 0.300 mm, 0.0750 mm, and 0.0374 mm sieve screens and then centrifuged at 1000 ×g for 10 min at RT to separate floating adipocytes from vascular-stromal cells. The precipitate was resuspended using Red Blood Cell Lysis Buffer (Solarbio, China) for 15 min at room temperature. After centrifugation at 1000×g for 10 min at room temperature, the pre-adipocytes were seeded at a density of 1×10^4^ cells/cm^2^ and cultured in a humidified atmosphere of 5% CO2 and 95% air at 37°C. The morphological features of pre-adipocytes were observed using a microscope (Olympus, Tokyo, Japan).

### Pre-adipocyte differentiation and oil red O staining

The basic medium was changed to the differentiation medium after achieving 90% cell confluence with 0.5 mmol/L IBMX, 1 μm/L DEX, 10 μg/μL insulin, 1 μM Rosiglitazone, 300 μM oleic acid added to the medium to induce pre-adipocyte differentiation for 3 days. The medium was replaced every second day. The differentiation medium was replaced with the maintenance medium of 10 μg/μL insulin for 2 days. The lipid droplets in pre-adipocytes were stained using an Oil Red O staining kit (Solarbio, China) according to the instructions. Briefly, cells were washed using PBS and fixed using 4% formaldehyde for 10 min. Then, the fixed cells were washed using PBS and stained with 1% filtered Oil Red O solution. After 30 min, the cells were repeatedly washed using distilled water and then observed using a microscope. For Oil Red O quantitative analysis, the intracellular adsorbed Oil Red O was extracted in 100% isopropanol for 10 min, and absorbance was measured at 500 nm wavelength.

### TG content analysis

Triglyceride (TG) content was analyzed using a triacylglycerol detection kit (Jiancheng, China) according to the manufacturer protocols (n = 3). Briefly, duck adipocytes were washed using preheated PBS and then were centrifuged at 1000 ×g for 5 min. The cells were ruptured using the ultrasonic cell-break method. A BCA protein assay kit (Beyotime, Beijing) was used to measure the protein concentration of ruptured cells. OD values were detected using a microplate spectrophotometer. TG content was expressed in mmol/g protein.

### RNA-seq analysis and circRNA library construction

Duck pre-adipocytes and those that had undergone differentiation for 3 days (n = 3) were collected for RNA-sequencing analyses of ribosomal RNA-depleted total RNA analyses. Thereafter, RNA libraries were constructed using the Truseq RNA-Seq Library Prep Kit v.2 (Illumina) following the manufacturer’s instructions. Libraries were sequenced using the Illumina HiSeq 2500 platform by paired-end sequencing, and this was completed by Novogene Biotechnology Co., Ltd. (Beijing, China).

### Sequence mapping, annotation, quantification, and verification of duck circRNAs

Sequence reads were first mapped using Bowtie [19] against the standard duck genome (BGI_duck_1.0 https://www.ncbi.nlm.nih.gov/assembly/GCA_000355885.1). Adapter reads and low-quality reads were removed from the raw sequencing data of each sample using Trim Galore. The find_circ package for python was applied to obtain back-spliced junction reads and detect GT-AG splicing signals for circRNA prediction [7]. The expression of circRNAs was quantified based on the number of reads spanning back-spliced junctions (circular reads). The total number of reads with back-spliced junctions was used to measure circRNA abundance, and Transcripts PerKilobase Million (TPM) was used to estimate the relative expression of each circRNA [20]. DE-circRNAs in two groups were identified using DESeq2 software with a NB test P-value < 0.05 and | log2 fold-change | > 0.5. Seven circRNAs (circRNA1044, circRNA1376, circRNA5347, circRNA8387, circRNA3172, circRNA7810 and circRNA595) were randomly selected for validation (Table 1). Considering the feasibility of further verification experiments, we made the criteria (at least two reads, length below 2000 nt, and a known host gene) refers to the relevant literatures [21,22]. Besides, three 7 day-old cherry valley ducks were sacrificed and seven tissues were collected for gene expression detection: heart, liver, spleen, lung, breast muscle, leg muscle, and adipose. All assays were run in triplicate and presented as the mean ± SEM. Relative circRNAs expression was calculated using the 2^−ΔΔCt^ method.

**Table 1:** Primers for verification circRNAs.

CircRNAs	Primers	
	Forward	Reverse
novel_circ_0001044	GTCAGACTGCACCATGAGCTG	AGCTCCTGCAGCTGGTAAACA
novel_circ_0001376	CGCTGTTCGTGGACAAGGAG	ACAAACCCAAACGAAGGGGG
novel_circ_0005347	TCATGGAGAGCGTAGTCCCG	GACAGCGCAACTCAAGTGGT
novel_circ_0008387	CAGGCATGGAGATTTTGGGC	TGGTCCTGAAGGAAAATTCAAGATG
novel_circ_0003172	AGAGCCGGTACCATGTGTCT	CTCGTGGTCCTCTGTCGCT
novel_circ_0007810	TGTGGAGCGCCTTTCACAAG	CACCTACCTTGCTGCAATCCT
novel_circ_0000595	GTTTGCAGGCAAAAAGGGGT	CCCACGTGTCTGCCATTTTC

### Circ-PLXNA1-miRNA-gene prediction and target gene functional analysis

To gain insight into the function of the circ-PLXNA1 in duck pre-adipocyte differentiation, the prediction of potential circRNA-miRNA-gene binding sites was performed using miRanda (http://www.microrna.org/microrna/getGeneForm.do). And gene ontology (GO) and Kyoto Encyclopedia of Genes and Genomes (KEGG) enrichment analyses were performed using DAVID 6.8 (http://david.abcc.ncifcrf.gov). CeRNA network was performed using Cytoscape 3.7.1 (https://cytoscape.org/download.html). A value of P < s0.05 was considered significantly enriched.

### Vector construction and oligonucleotide transfection

Short interfering RNAs (siRNAs) against circ-PLXNA1 (5’-GGGAAGATCAAAAAGGGCT-3’), CTNNB1 (5’-CACTAACCAAGCTGAGTTT-3’), miR-214 mimics (5’-GACGGACAGACACGGACGACA-3’) and negative controls (si-NC and miRNA-NC) used for cell transfection were synthesized by Ribobio Co., Ltd. (Guangzhou, China). The 100 nM siRNAs and 50 nM miRNAs were transfected into pre-adipocytes using the Lipofectamine2000 Reagent (Life Technologies). A luciferase reporter with the included target site of the circ-PLXNA1 or CTNNB1 was constructed by subcloning the fragment into the region directly downstream of a cytomegalovirus promoter-driven firefly luciferase cassette in the pMIR-report vector (Promega). Mutations of both miR-214-binding sites were synthesised by Sangon Co., Ltd. (Shanghai, China). Primer sequences are shown in Table 2. All constructs were verified by Sanger sequencing.

**Table 2:** Primer sequences for PCR.

Primers	Sequence (5’-3’)	Product length
Duck circ-PLXNA1 divergent primer	TGTTCGTGGACAAGGAGGAC	185bp
	AGAGGAGCTAACGGGACACA	
Duck circ-PLXNA1 convergent primer	TGTGTCCCGTTAGCTCCTCT	1389bp
	GTCCTCCTTGTCCACGAACA	
PLXNA1	GCTGCCCTGGCTCTTGAA	160bp
	CACCGAGGTCATCCCGTC	
LPL	GAGGGAACCTGATTCAAACG	124bp
	CATCCAGTCAATAAACATAGCG	
DLK1	CTGTGCCCTTCTGGTTTTGC	192bp
	TCCATTCTCACATGGGCCAC	
C/EBPα	GTGCTTCATGGAGCAAGCCAA	191bp
	TGTCGATGGAGTGCTCGTTCT	
PPARγ	CCCAAGTTTGAGTTCGCTGT	196bp
	GCTGTGACGACTCTGGATGA	
FABP4	AATGGCTCACTGAAGCAGGT	143bp
	TGGCTTCTTCATGCCTTTTC	
FAS	TGGGAGTAACACTGATGGC	109bp
	TCCAGGCTTGATACCACA	
CTNNB1	GTCACCTCACCAGCAGACAC	202bp
	AGCCTTGGAATAGCACCTTG	
β-actin	ATGTCGCCCTGGATTTCG	165bp
	CACAGGACTCCATACCCAAGAA	
GAPDH	CACAGCCACACACGAAGACA	106bp
	CCTTAGCCAGCCCCAGTAGA	
circ-PLXNA1-sac1-F	cgagctc-GACTGTGTCCCGTTAGCTCC	341bp
circ-PLXNA1-hind3-R	cccaagctt-GAGCGTCAGGTTGTTGGAGA	
CTNNB1-sac1-F	cgagctc-AAGCAGGTGGATCTATTTCATGT	176bp
CTNNB1-hind3-R	cccaagctt-ACTGCATTTTTCTCATGAAGCAT	

### Reverse transcription and quantitative real-time PCR (qRT-PCR)

Total RNAs were extracted from the cultured cells using Trizol reagent (Invitrogen, USA). Rnase R (Epicenter) was used to digest linear RNAs at 37℃ for 15 min (3U Rnase R/μg RNA). Nuclear and cytoplasmic extraction reagents (Thermo Scientific) was used to elucidate the subcellular distribution of circ-PLXNA1. First-strand cDNA was synthesized from 1 μg of total RNA using a cDNA synthesis kit following the manufacturer instructions (TaKaRa, Japan). The mRNA expression levels of several marker genes for pre-adipocyte differentiation were detected using a SYBR PrimerScript^TM^ RT-PCR kit (TaKaRa, Japan) and the QuantStudio 5 real-time PCR instrument. GAPDH, β-actin or U6 as internal controls and all assays were run in triplicate. Mir-X™ miRNA First-Strand Synthesis (TaKaRa, Japan) and TB Green™ qRT-PCR User Manual (TaKaRa, Japan) for the miR-214 qRT-PCR (n = 3). Primer sequences used are listed in Table 2. Relative transcription alteration was evaluated as 2^-ΔΔCt^.

### Dual-luciferase assay

293T cells provided by Professor Song from Yangzhou University in China were seeded into 24 well plates at a density of 1×10^5^ cells/well, and incubated at 37°C overnight. Cells were transfected with reporter plasmids (100 ng) in the presence of miR-214 mimics or its control miRNA-NC along with PRL-TK (Promega) (5 ng) by using Lipofectamine 2000 (Invitrogen). Luciferase activity was measured after 24 h using a dual-luciferase reporter assay detection kit (Promega) (n = 6). Firefly luciferase activities were normalized to Renilla luciferase activity. Finally, the foldchange was calculated by miR-214 compared with miRNA-NC.

### Western blot analysis

Total protein was extracted from cells using a RIPA buffer (Beyotime) supplemented with a protease inhibitor Cocktail (BioDee) (100:1). Protein was separated on 8% SDS-PAGE gels (GenScript). The proteins were transferred to PVDF membrane, and then blocked with 5% non-fat milk for 1 h. The membranes were incubated with the primary antibodies CTNNB1 (1:1000, NBP1-54467SS, Novus) and GAPDH (1:5000, AP0066, Bio-world) for 1h and washed with PBST (Solarbio) three times (5 min/time). Then the membranes were incubated with anti-mouse (1:3000, ab6728, Abcam) or anti-rabbit (1:3000, 7074P2, CST) secondary antibody conjugated with HRP for 1 h at room temperature and washed three times using PBST. Protein bands were visualized using enhanced chemiluminescence (ECL) system (GE Healthcare, USA) and quantified with ImageJ Software, as compared to GAPDH.

### Statistical analysis

Statistical analysis was performed using GraphPad Prism 6 Software and SPSS 22.0. Meaningful differences between the groups were calculated using a Student's t-test. P < 0.05 was considered statistically significant.

## Results

### Duck pre-adipocyte differentiation

**Fig 1:**
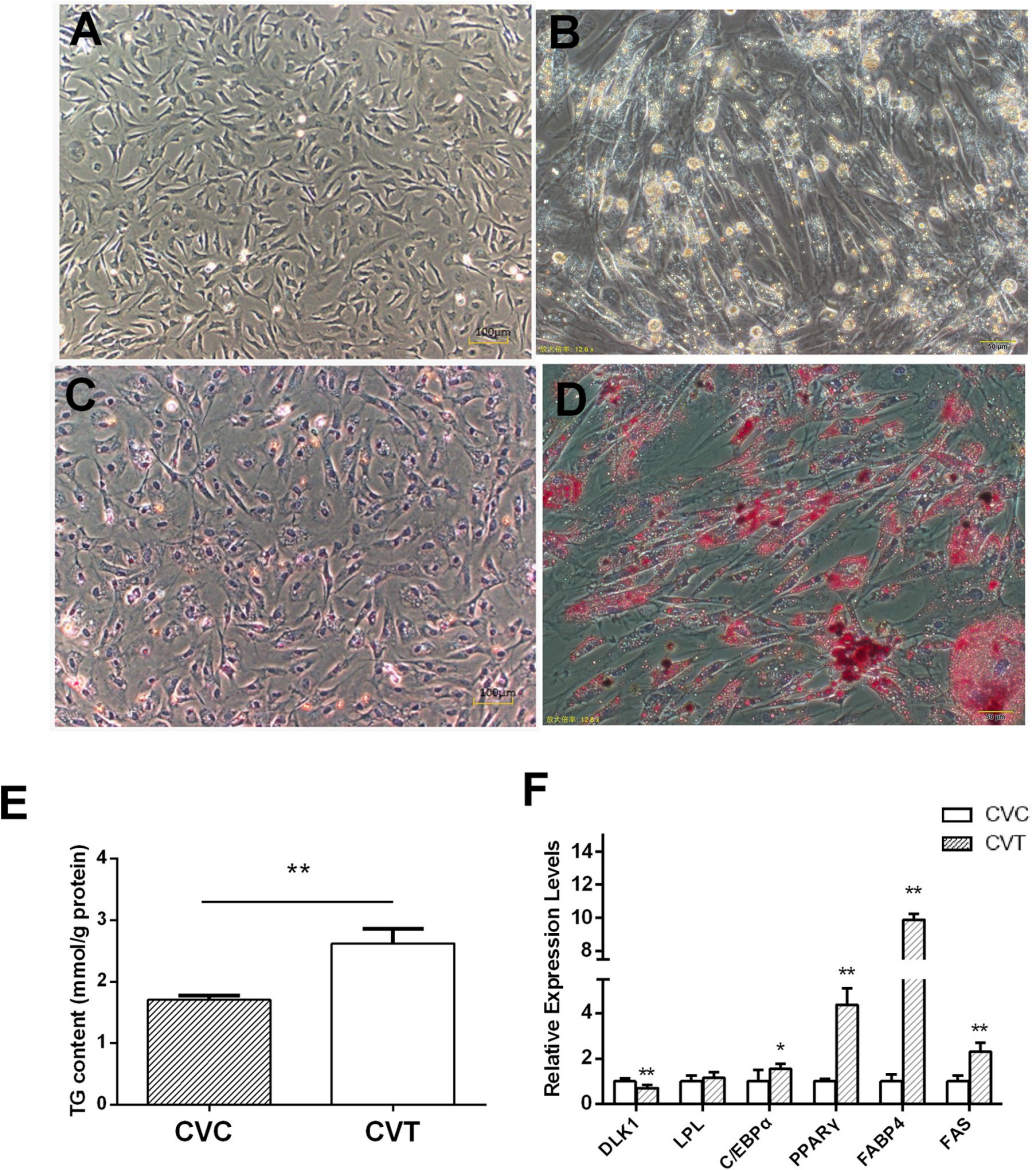
In vitro adipogenesis of primary subcutaneous pre-adipocytes. **(A)** Cellular morphology of CVC. **(B)** Cellular morphology of CVT. (**C)** Oil red staining of CVC. (D) Oil red staining of CVT. **(E)** Triglyceride content in CVC and CVT. **(F)** The relative mRNA expression of six genes related to differentiation and adipogenesis in duck adipocyte. *P < 0.05, ** P< 0.01.

The morphological characteristics of cherry valley duck primary pre-adipocytes control group (CVC) and cherry valley duck pre-adipocytes treated by 72 h differentiation (CVT) are shown in Fig 1A–1D. The cells presented a fusiform morphology with lipid droplets filling the center of the cells. The lipid droplets in these cells were stained with oil red O. The triglyceride (TG) content in CVT was significantly higher than CVC (Fig 1E). After differentiation, all of the marker genes, including DLK1, LPL, C/EBPα, PPARγ, FABP4, and FAS were detected in the culture of adipocyte cells (Fig 1F). The expression levels of C/EBPα, PPARγ, FABP4, and FAS were increased and DLK1 was decreased in differentiated adipocytes compared to the pre-adipocytes, which showed normal differentiation progress.

### Profiling of circRNAs in duck pre-adipocytes after differentiation

**Fig 2:**
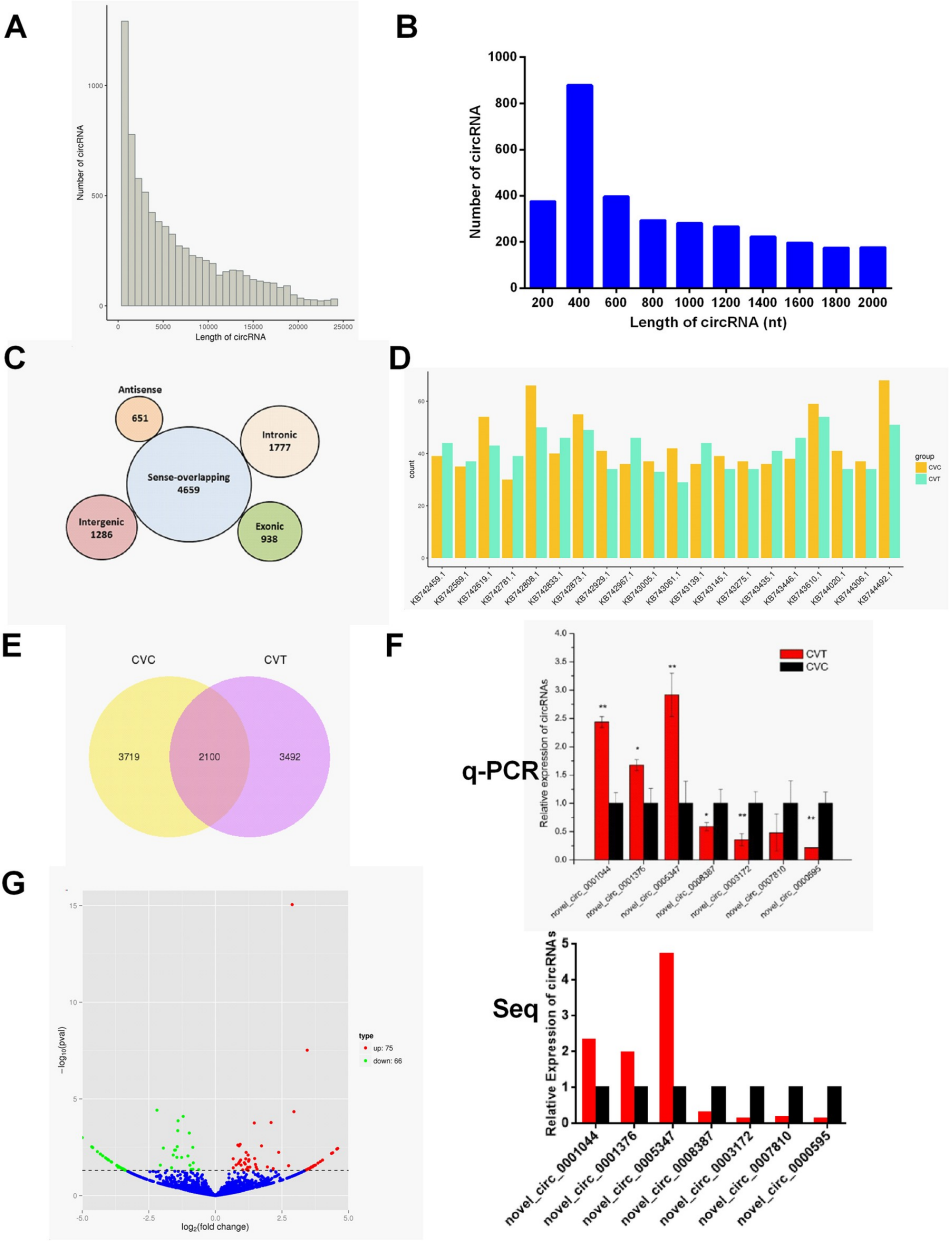
Overview of circRNA expression in CVC and CVT. **(A)** Sequence length distribution of all circRNAs. **(B)** Identified circRNAs with sequence lengths distribution < 2000 nt. **(C)** The proportion of different circRNAs among all predicted circRNAs. **(D)** Numbers of identified circRNAs in different positions of duck genomes. **(E)** Venn diagrams showing the numbers of common and unique circRNAs between CVC and CVT. **(F)** Comparison of qRT-PCR and sequencing results in adipocytes differentiation. **(G)** Volcano Plot of DE-circRNAs comparing CVC and CVT.

To generate a circRNA profiling database, we performed RNA-seq analyses of RNA from CVC and CVT (n = 3). A total of 314,419,642 and 288,772,480 raw reads were generated from the CVC and CVT libraries, respectively (S1 Table). After removing adapter reads and low quality reads using Trim Galore, we identified 301,041,712 and 275,667,250 clean reads, respectively, for further analysis. In total, we identified 9,311 circRNA candidates in duck adipocytes using the find_circ package and the length distribution of circRNAs are shown in Fig 2A and 2B. We focused on the circRNAs that were less than 2000 nucleotides (nt) in length. Most of them were 400 nt with an average length of 760 nt. Approximately half of all circRNAs (50.04%) were sense-overlapping circRNAs, while a small proportion of circRNAs (10.07%) were exonic circRNAs (Fig 2C). The genome distribution of identified circRNAs did not reveal any obvious differences between CVC and CVT (Fig 2D). There were 2,100 common circRNAs in the CVC and CVT groups, and the total expression number of circRNAs in CVT was downregulated (Fig 2E). Seven randomly-selected circRNAs were validated by fluorescent qRT-PCR. The results of three independent repetitions of qRT-PCR were highly consistent with the RNA-seq results (Fig 2F).

### Differentially expressed circRNAs in duck adipocytes differentiated for 3 days

**Fig 3:**
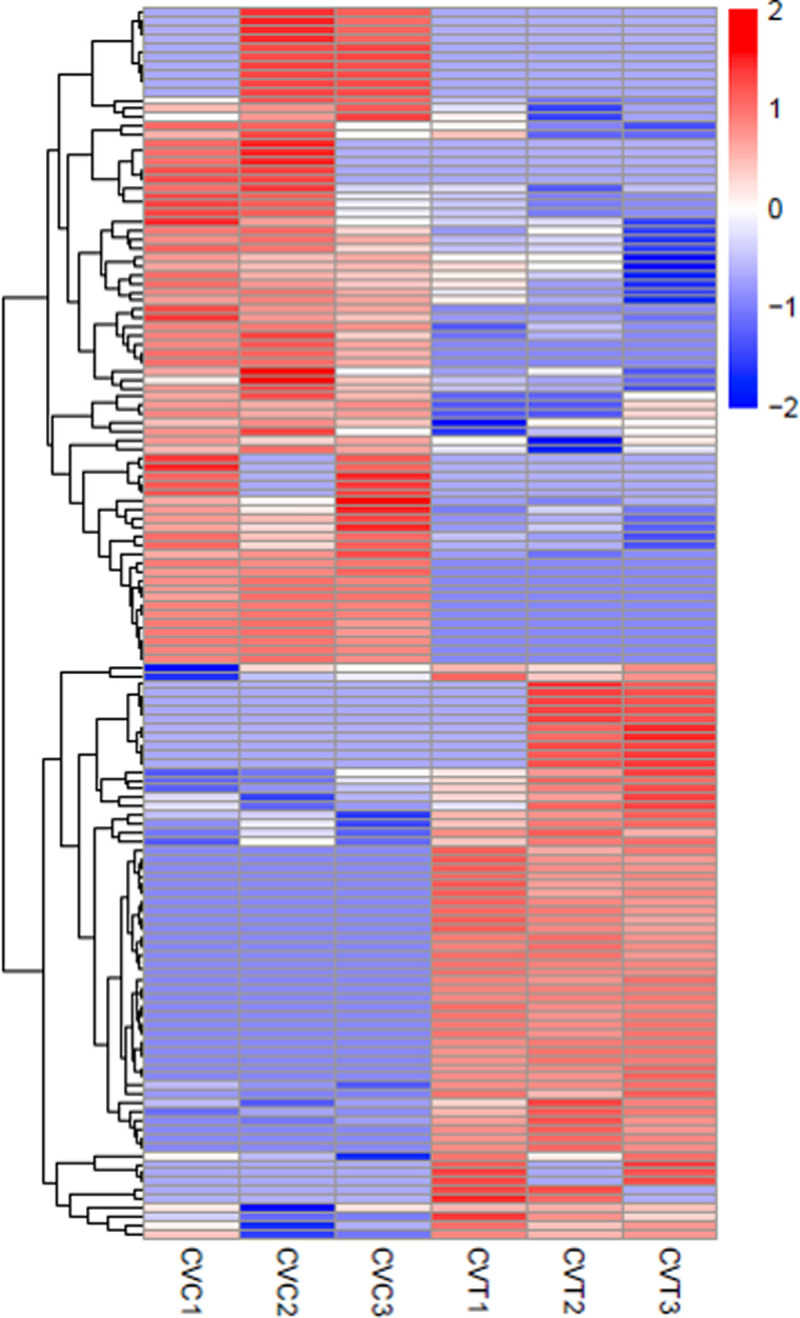
Heat map of all DE-circRNAs between CVC and CVT.

**Fig 4:**
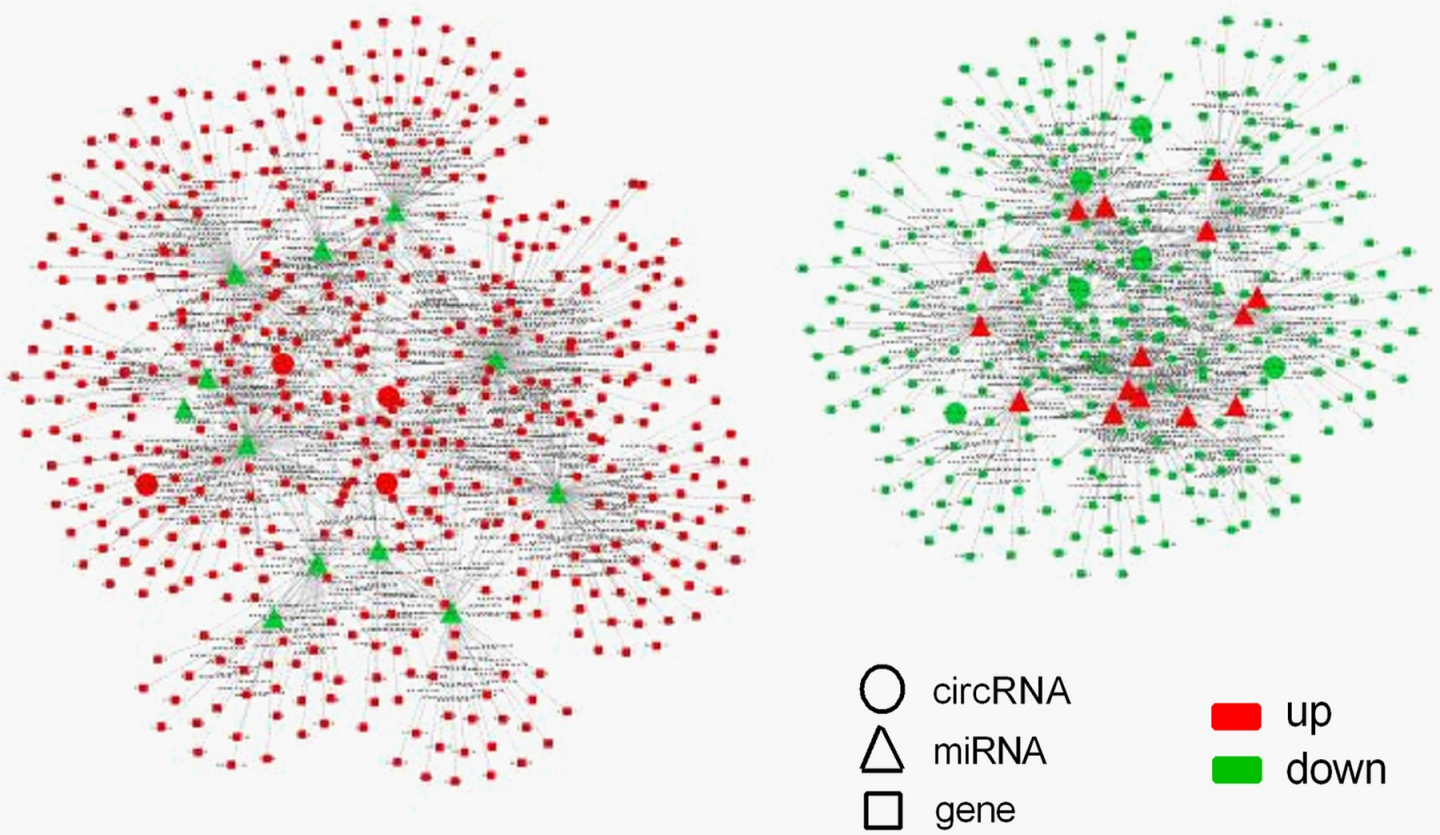
Competing endogenous-RNA network exhibited by 10 selected circular-RNAs.

A volcano plot showed the different expression patterns of circRNAs (Fig 2G). Heat maps were generated to illustrate the 141 significantly DE circRNAs in CVT compared to CVC (Fig 3). 75 were up-regulated and 66 were down-regulated circRNAs in CVT relative to CVC. Only 11 circRNAs matched the specified search terms (Table 3). Cytoscape was combined with the sequencing results to demonstrate the ceRNA network involved 10 circRNAs, 27 miRNAs and 808 mRNAs except for circ-8371 which had no target miRNA (Fig 4).

**Table 3:** Overview of 11 DE-circRNAs matching the filter criteria.

circRNA	full length(bp)	feature	Gene Name	CVT readcount	CVC readcount	log2Fold Change	P value
circ-0365	1948	exon9,exon10	ARHGAP21	24.234	41.462	-0.779	0.026
circ-1044	1917	exon1,intron1,exon2	MTCL1	157.590	68.139	1.210	0.000
circ-1376	1534	5’UTR,exon1,exon2	PLXNA1	111.861	56.849	0.980	0.001
circ-1526	389	exon1	METRNL	3.052	9.441	-1.565	0.044
circ-3377	1468	exon1	FBLN2	57.158	107.022	-0.889	0.003
circ-5802	856	exon1	SPSB4	10.320	22.077	-1.087	0.020
circ-6050	1740	exon1	ZBTB1	9.585	22.490	-1.226	0.008
circ-6443	258	exon5	MBNL1	17.628	32.406	-0.870	0.022
circ-8371	606	exon14	AHNAK	10.647	23.998	-1.140	0.013
circ-8614	1680	exon24,exon25,exon26	MAPKBP1	69.207	44.276	0.626	0.045
circ-8890	414	exon11	LCMT2	141.962	78.520	0.849	0.003

### Circ-PLXNA1 identification and expression patterns in ducks

**Fig 5:**
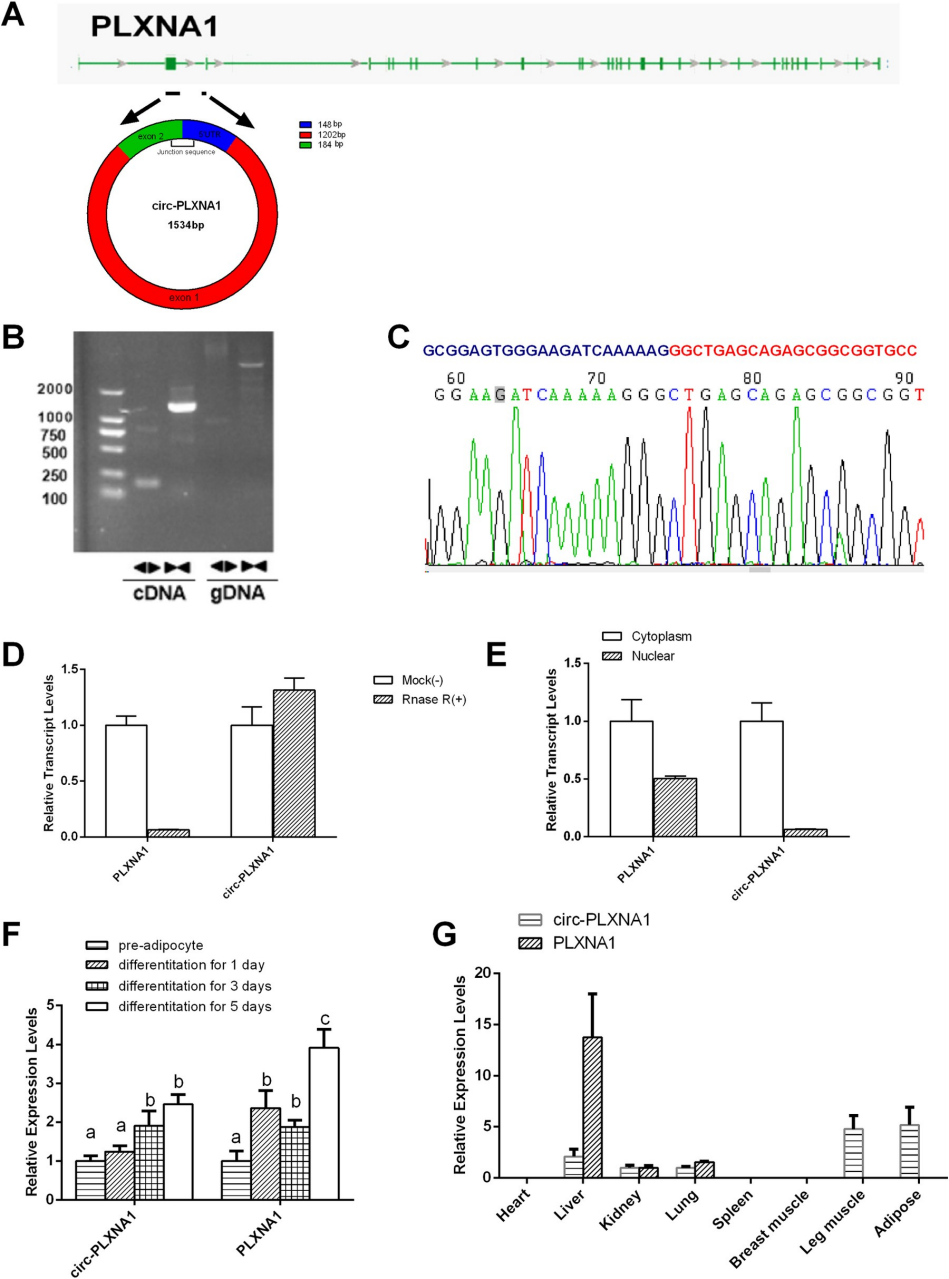
Characterization of circ-PLXNA1 in duck adipocyte. **(A)** The genomic loci of circ-PLXNA1 in PLXNA1 gene. **(B)** The validation strategy for circ-PLXNA1. Clear single bands were amplified from the cDNA of duck pre-adipocyte using divergent primers. Clear single bands could not be amplified from gDNA. **(C)** Sanger-Seq validated the head-to-tail junction region of duck pre-adipocyte circ-PLXNA1. **(D)** qRT–PCR result for the abundance of circ-PLXNA1 and PLXNA1 mRNA in duck adipocyte treated with RNase R. **(E)** The subcellular distribution of circ-PLXNA1. Nuclear and cytoplasmic RNA was extracted, and junction primers were used for circ-PLXNA1 detection. **(F)** The transcripts level of circ-PLXNA1 and PLXNA1 during duck adipocyte differentiation. The different letters show significant differences in common gene, P < 0.05. **(G)** The relative expression level of circ-PLXNA1 in seven duck tissues by qRT-PCR.

We focused on the candidate that had the high expression level and extensive regulatory network. Among these specific candidates, circ-1376, which had the greatest number of potential target miRNAs, attracted our attention. Circ-1376 is derived from the spliced transcript of PLXNA1 and is composed of part of the 5’UTR, exon 1 and exon 2 (Fig 5A). This form is renamed circ-PLXNA1. Class A plexins (PLXNAs) act as semaphorin receptors and control diverse aspects of nervous system development and plasticity, ranging from axon guidance and neuron migration to synaptic organization [23]. It has been shown that circRNAs form in a human hepatic stellate cell line [24] and drosophila cells [25], though their sequences are different. We monitored the circRNA-characteristic splice junction sequence and the linear sequence by amplifying it from the cDNA of duck pre-adipocytes, using the divergent/convergent primer strategy, while the circRNA-characteristic splice junction sequence was not amplified by genomic DNA (Fig 5B). Furthermore, the amplified product of circ-PLXNA1 was sequenced using Sanger-sequencing to validate the circularized junction of the circRNAs (Fig 5C). After digestion by RNase R, the linear PLXNA1 RNA transcripts were significantly more degraded compared with circ-PLXNA1, further validating the proposed circular nature of the circ-PLXNA1 transcript (Fig 5D). Next, we used qRT-PCR in cell fractions to elucidate the subcellular localization of circ-PLXNA1 in duck pre-adipocytes. After the separation of cytoplasmic from nuclear material, qRT-PCR showed that circ-PLXNA1 was mainly localized in the cytoplasm (Fig 5E). The expression of circ-PLXNA1 in adipocytes increased linearly with the number of cultured days in differentiation medium, while such expression levels did not correlate with the expression of host gene PLXNA1 (Fig 5F). Circ-PLXNA1 is mainly expressed in adipose, leg muscle, and the liver and no expression was found in the heart, spleen and breast muscle. Linear PLXNA1 mRNA is mainly expressed in the heart (Fig 5G).

### Circ-PLXNA1 levels affected during duck pre-adipocytes differentiation

**Fig 6:**
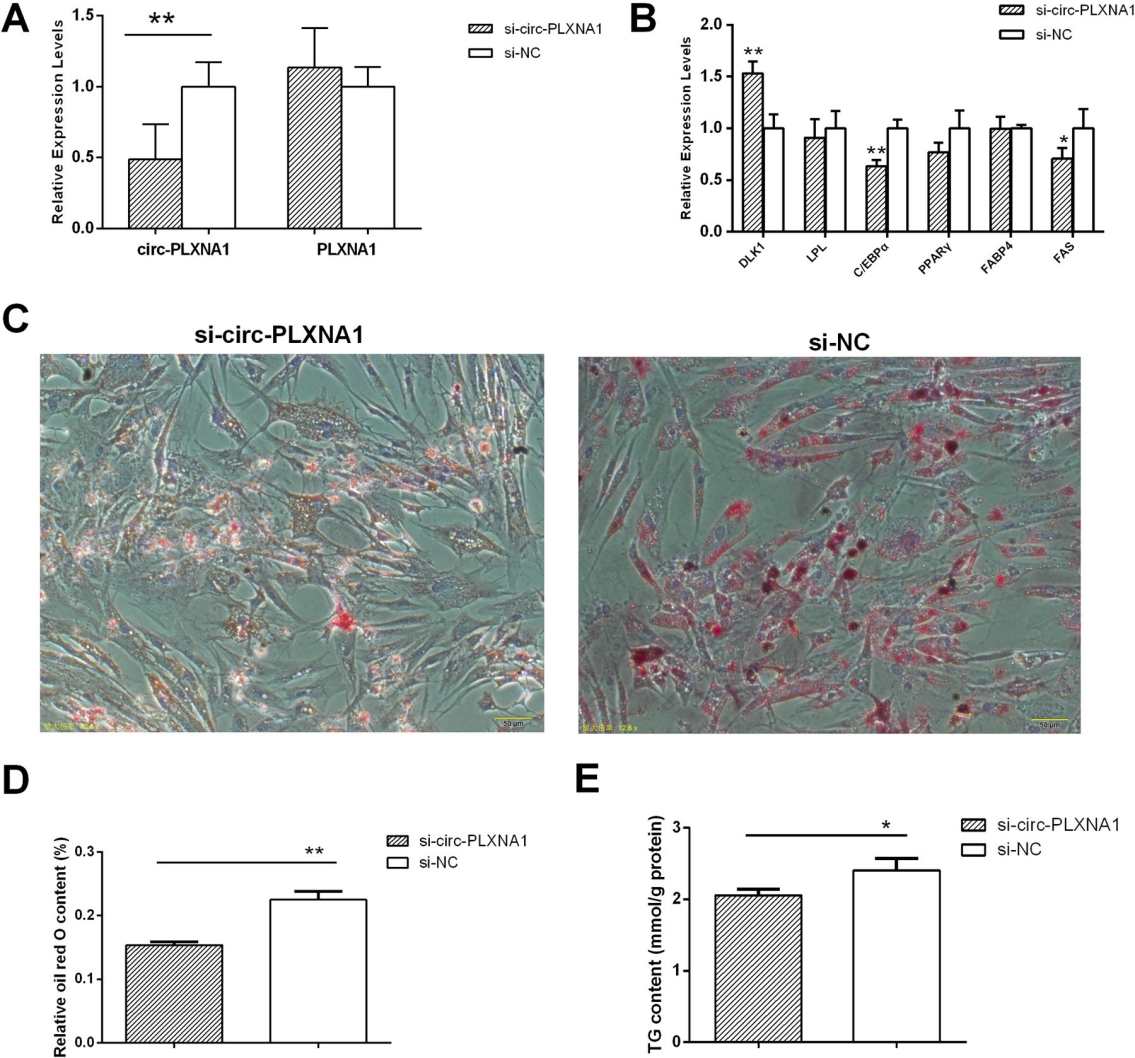
Affection of si-circ-PLXNA1 in pre-adipocytes differentiation. **(A)** The inhibition effects of si-circ-PLXNA1 on the transcripts level of circ-PLXNA1 and PLXNA1. **(B)** The relative mRNA expression of six genes related to differentiation and adipogenesis in duck adipocytes affected by si-circ-PLXNA1. **(C)** Duck adipocytes morphology stained by oil red O after the transfection of si-circ-PLXNA1 or si-NC and 72 h differentiation. **(D)** The oil red O content by spectrophotometric analysis. **(E)** TG content in duck adipocytes affected by si-circ-PLXNA1. *, P < 0.05, **, P< 0.01.

To fully understand the function of circ-PLXNA1 in adipocyte differentiation, we further synthesized circ-PLXNA1 siRNA according to the junction sequence. The new siRNA displayed maximal inhibition of 51.3% and was selective, leaving PLXNA1 levels the same as baseline (Fig 6A). The expression profiles of the six marker genes affected by circ-PLXNA1 knockdown are shown in Fig 6B. C/EBPα and FAS are significantly down-regulated, and DLK1 is significantly increased. When cells transfected with si-circ-PLXNA1 were differentiated to day 3, the number of lipid droplets (Figs 6C and S1), the adipogenic capacity of preadipocytes (Fig 6D) and the TG content (Fig 6E) were reduced compared to normal differentiated adipocytes. Together, the data suggest that circ-PLXNA1 may act as a promoter in duck pre-adipocytes differentiation.

### Circ-PLXNA1 function patterns predicted by bioinformatic analysis

**Fig 7:**
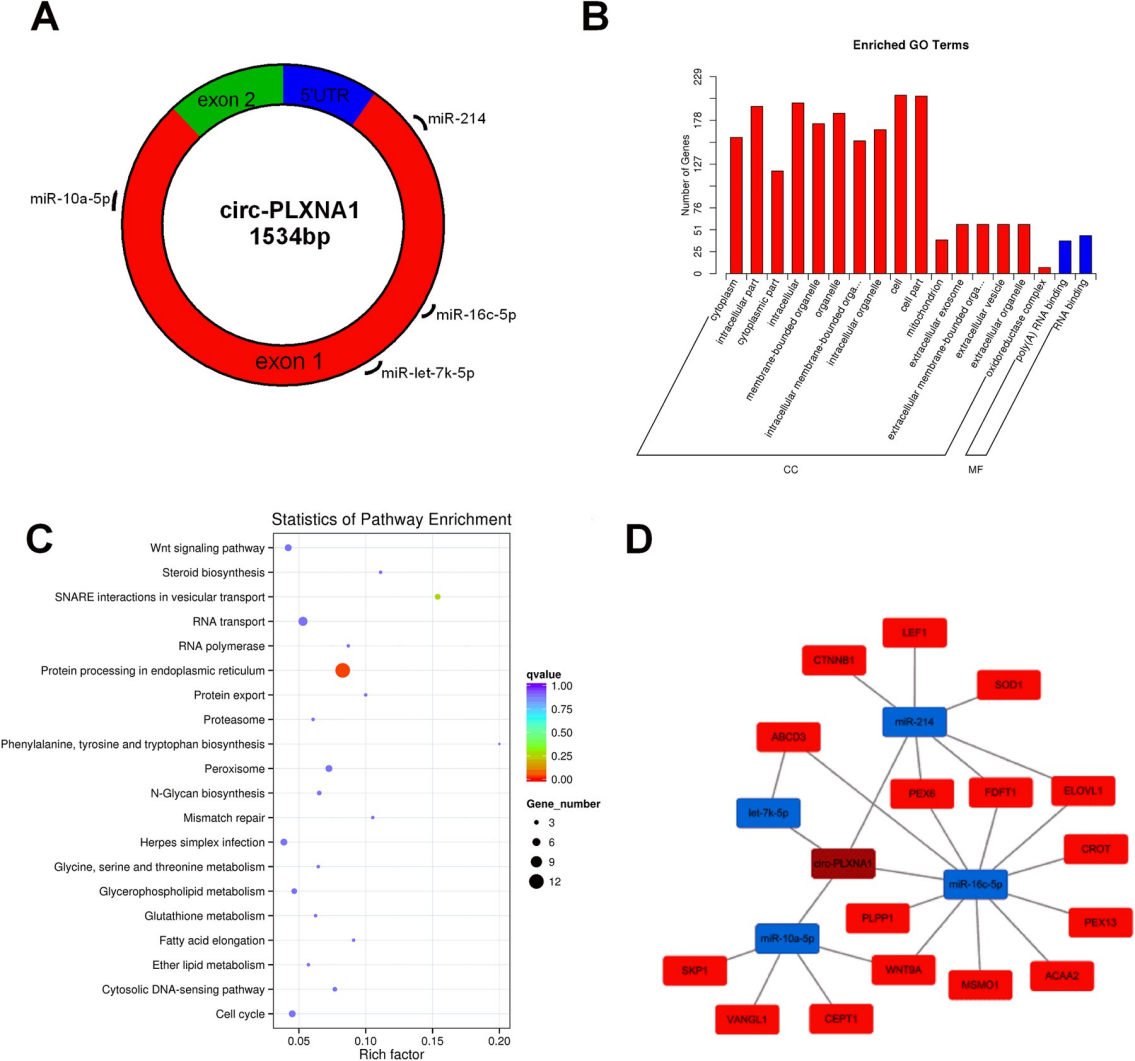
Bioinformatics analysis of circ-PLXNA1. **(A)** Predicted binding sites of four miRNAs in circ-PLXNA1. **(B)** Top 18 GO terms in each category of 313 target genes. **(C)** Top 20 KEGG signaling pathway annotations of 313 target genes. **(D)** CeRNA network of circ-PLXNA1, four miRNAs and 16 mRNA involved in four pathways (peroxisome, steroid biosynthesis, fatty acid elongation, Wnt signaling pathway, ether lipid metabolism), blue frames for down-regulated genes, red frames for up-regulated miRNAs.

The results of transcriptome sequencing were also used to elucidate possible functions for circ-PLXNA1 during duck pre-adipocyte differentiation. Four miRNAs (let-7k-5p, miR-10a-5p, miR-16c-5p, miR-214) were predicted to target circ-PLXNA1 (Fig 7A) and 313 genes were predicted to target those four miRNAs analyzed by miRanda program. GO enrichment analysis of target genes revealed 18 significantly enriched GO terms, which were mainly related to “cellular components” (Fig 7B). KEGG pathway enrichment analysis showed significant enrichments in protein processing in the endoplasmic reticulum, RNA transport, peroxisome, cell cycle and Wnt signaling pathway (Fig 7C). Sixteen target genes from four pathways (peroxisome, steroid biosynthesis, fatty acid elongation, the Wnt signaling pathway, and ether lipid metabolism) related to adipocyte differentiation and lipid metabolism were selected to curate a ceRNA network (Fig 7D). It is important to note that cadherin-associated protein β1, encoded by CTNNB1, is an important gene involved in the Wnt signaling pathway which plays a key function during the differentiation of many cell types [26–28]. Therefore, we speculated that circ-PLXNA1/miR-214/CTNNB1 might represent a key functional axis for duck pre-adipocyte differentiation.

### Target verification among circ-PLXNA1/miR-214/CTNNB1

**Fig 8:**
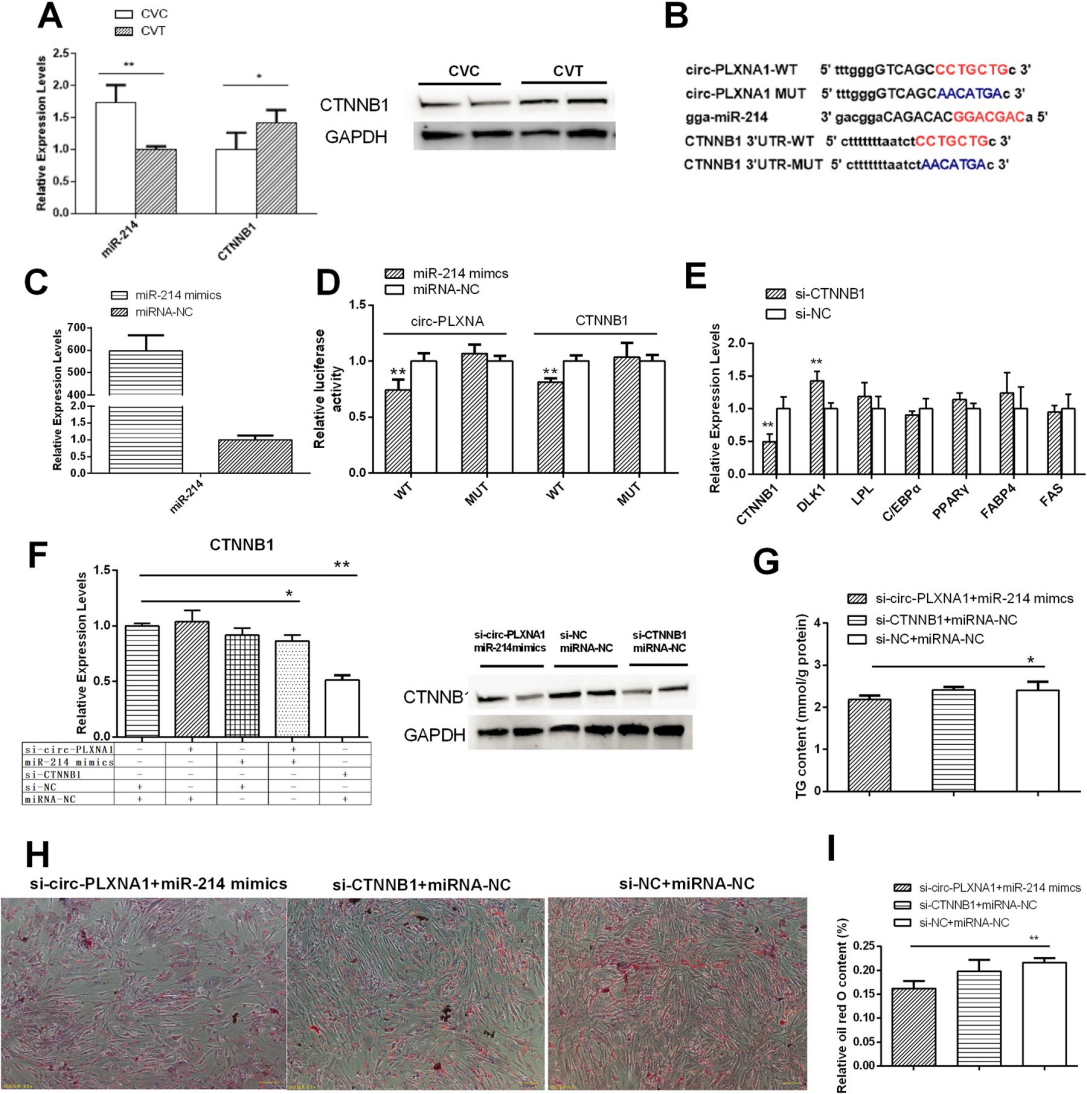
Circ-PLXNA1/miR-214/CTNNB1 axis in duck adipocytes differentiation. **(A)** The expression patterns of miR-214 and CTNNB1 in CVC and CVT. **(B)** Predicted binding sites and mutation sites in circ-PLXNA1 and CTNNB1 3’UTR with miR-214 by bioinformatics analysis. **(C)** The efficacy of miR-214 mimics. **(D)** Verification of miR-214 target circ-PLXNA1 and CTNNB1 3’UTR using luciferase assay. **(E)** Differentiation marker genes expression level after si-CTNNB1 treatment in duck pre-adipocytes compared to the control cells. **(F)** Expression levels of CTNNB1 affected by combination transfection. **(G)** Triglyceride content in duck adipocytes transfected with si-CTNNB1 or si-circ-PLXNA1 and miR-214 mimics for 48 h and differentiation for 3 d. **(H)** Oil Red O staining by duck adipocytes transfected with si-CTNNB1 or si-circ-PLXNA1 and miR-214 mimics for 48 h and differentiation for 3 d. **(I)** The oil red O content by spectrophotometric analysis. *P < 0.05, **P < 0.01.

The expression of miR-214 was inversely correlated with that of circ-PLXNA1 and CTNNB1 in adipocyte differentiation (Figs 5F and 8A). MiRanda analysis indicated that there was a complementary sequence in circ-PLXNA1 for miR-214, and miR-214 could also target the 3’-UTR of CTNNB1 (Fig 8B). The overexpression efficiency of miR-214 mimics was good (Fig 8C). A dual-luciferase reporter assay was used to confirm the interaction between circ-PLXNA1 and miR-214, or miR-214 and CTNNB1. The results showed that miR-214 overexpression significantly inhibited the luciferase activity of the circ-PLXNA1 reporter and CTNNB1 3’UTR (Fig 8D), suggesting there was a direct interaction between circ-PLXNA1 and miR-214 or miR-214 and CTNNB1. Si-CTNNB1 had an interference efficiency of 50.0%. The inhibition of CTNNB1 led to the up-regulation of DLK1 and no significant changes in other differentiation marker genes compared to the negative control group (Fig 8E). Furthermore, the expression level of CTNNB1 was decreased by co-transfection of si-circ-PLXNA1 and miR-214 mimics, adding further support to the possibility that CTNNB1 was a target of miR-214 (Fig 8F). The TG levels and the adipogenic capacity of preadipocytes decreased upon co-transfection of si-circ-PLXNA1 and miR-214 mimics, while they changed a little after si-CTNNB1 transfection (Fig 8G, 8H and 8I). The result suggested that circ-PLXNA1/miR-214/CTNNB1 might exist in duck adipocyte, however, it had a limited function in adipocyte differentiation, circ-PLXNA1 and miR-214 may play more important roles in adipocyte differentiation.

## Discussion

Successful systems for the isolation, culture, and induction of duck primary fat cells have previously been developed [2,5]. After 72 h of culture in differentiation medium, small lipid droplets gradually accumulated into large lipid droplets inside cells, TG content was significantly increased, and the expression of differentiation marker genes was consistent with previous reports [2]. Transcriptomics data presented here showed that 9,311 circRNA candidates were identified and half of them were sense-overlapping circRNAs. There were 141 DE-circRNAs in CVC and CVT. Ten circRNAs were selected to draw the ceRNA network diagram which involved 27 miRNAs and 808 mRNAs. These circRNAs and miRNAs may play an important role in the complex gene regulation network during adipocyte differentiation. In hepatocellular carcinoma (HCC) 15 circRNAs, 17 miRNAs, and 89 mRNAs were found to be correlated in ceRNA network [29]. Previous studies have explored the expression profiles of non-coding RNAs (ncRNAs) including miRNAs and long non-coding RNAs (lncRNAs) in poultry [30,31], however, the specific function of circRNAs in duck adipocytes is still unknown.

We investigated a circRNA called circ-PLXNA1 that is transcribed from the PLXNA1 gene in duck adipocytes. Duck circ-PLXNA1 is mainly localized in the cytoplasm and expressed in adipose, leg muscle, and liver. In avian species, de novo fatty acid synthesis takes place mainly in the liver, lipids may be stored in adipocytes, hepatocytes and muscles [32]. As the expression level of circ-PLXNA1 correlated to differentiation stages of duck adipocytes and it mainly expressed in tissues relative to fat, we speculated that circ-PLXNA1 may play an important role in duck adipogenesis. Circ-PLXNA1 expression is unlikely to be correlated with the linear PLXNA1 mRNA. Many plexin-A1 variants present in human [33] and several circRNAs formed by PLXNA1 have been detected in *Caenorhabditis elegans* [25], although its function has not yet been reported. The inhibition of circ-PLXNA1 up-regulated the expression level of DLK1 and down-regulted the expression levels of C/EBPα and FAS, which play important roles during duck adipogenesis. Transcriptional repression of C/EBPα was proved to inhibit adipocyte differentiation and impaired the accumulation of triglyceride [34]. FAS is a critical metabolic enzyme for lipogenesis, and it catalyzes the synthesis of saturated fatty acids in cells [35]. The result indicated that circ-PLXNA1 functioned during duck adipocyte differentiation.

There are four miRNAs targeted by circ-PLXNA1 and 313 target genes that bind to those miRNAs. We evaluated the function of circ-PLXNA1 from the perspective of those target genes, most of which were related to “cellular components”. Four pathways related to adipocyte differentiation and lipid metabolism were selected to focus on the circ-PLXNA1/miR-214/CTNNB1 axis. The expression of miR-214 was inversely correlated with that of circ-PLXNA1 and CTNNB1 in adipocyte differentiation. Accumulated evidence has shown that circRNAs act as miRNA sponges and regulates their function. For instance, the circ-HIPK3 transcript acts as a microRNA-124 (miR-124) sponge and regulates the expression of miR-124 mRNA targets, including SphK1, CDK4, and STAT3, in lung cancer cells [36]. Circ-NCX1 levels were shown to increase in response to reactive oxygen species (ROS) and promoted cardiomyocyte apoptosis by acting as an endogenous miR-133a-3p sponge [37]. Besides this, it was also reported that circ-MTO1 suppresses hepatocellular carcinoma progression by acting as a sponge for miR-9 in hepatocellular carcinoma tissues [38].

The duck adipocyte has a lower expression level of miR-214 and higher expression level of circ-PLXNA1 and CTNNB1 after differentitation. miR-214 is a deregulated miRNA in many pathological conditions [39] and could affect many biological processes [40,41]. miR-214 may serve as a diagnostic biomarker for obesity and insulin resistance [42]. Our dual-luciferase reporter experiment verified that miR-214 was likely able to suppress CTNNB1 by interacting with the 3' untranslated region of the CTNNB1 gene and that circ-PLXNA1 also could suppress miR-214 by interacting with the target site. Inhibition of CTNNB1 up-regulated the expression of DLK1 and no obvious change of the expression levels of other genes. After co-transfection of adipocytes with si-circ-PLXNA1 and miR-214 mimics or si-CTNNB1, CTNNB1 expression level were significantly decreased. MiRNAs control gene expression by regulating mRNA stability and translation [43]. MiR‑214 was demonstrated to directly interact with the 3'‑UTR of the β‑catenin gene CTNNB1 during osteogenic differentiation of periodontal ligament stem (PDLSC) [44]. And miR-214 was also demonstrated to target CTNNB1 in human cervical and colorectal cancer cells [45] and 3T3-L1 pre-adipocyte [46]. Our result primarily verified circ-PLXNA1/miR-214/CTNNB1 in duck adipocyte but had little influence on triglyceride content and the adipogenic capacity of preadipocytes. However, combination of circ-PLXNA1 and miR-214 might function during duck adipocyte differentiation. We speculated that there should be other mechanism involved in circ-PLXNA1 function, for example circRNAs may work by encoding peptides or proteins [47], circ-PLXNA1 may regulate other genes by target miR-214. Besides, the process of duck adipocyte differentiation may be difficult to analyse owing to complex network mechanisms.

## Conclusions

To the best of our knowledge, our study represents the first analysis of circRNAs expression profiles during the differentiation of pre-adipocytes in the subcutaneous fat of ducks. The lack of a circRNA database for poultry has limited the progress of such research. We verified circ-PLXNA1 in duck adipocytes and mainly expressed in duck adipose, leg mucle and liver. Our research suggested that inhibition of circ-PLXNA1 limited the differentiation of duck adipocyte. However, ceRNA“circ-PLXNA1/miR-214/CTNNB1 axis” exhibited limited function during differentiation. Further study should be done to clarify the function mechanism of circ-PLXNA1. Altogether, the circRNAs profiling and the role of circ-PLXNA1 should provide a basis for insights into the mechanisms of duck adipocyte differentiation.

## Supporting information

S1 Fig (A, B) Oil Red O staining by duck adipocytes transfected with si-circ-PLXNA1 for 48 h and differentiation for 3 d. (C, D) Oil Red O staining by duck adipocytes transfected with si-NC for 48 h and differentiation for 3 d.
(DOCX)

S2 Fig (A, B) Oil Red O staining by duck adipocytes transfected with si-circ-PLXNA1 and miR-214 mimics for 48 h and differentiation for 3 d. (C, D) Oil Red O staining by duck adipocytes transfected with si-CTNNB1 and miRNA-NC for 48 h and differentiation for 3 d. (E, F) Oil Red O staining by duck adipocytes transfected with si-NC or miRNA-NC for 48 h and differentiation for 3 d.
(DOCX)

S1 Raw images (PDF)

S1 Table Overview of sequencing data. (DOCX)
