# Supplementary material for: Circular RNA expression profiling reveals that circ-PLXNA1 functions in duck adipocyte differentiation
Source: PLoS One. 2020 Jul 21;15(7):e0236069. doi: 10.1371/journal.pone.0236069 (PMC7373283; doi:10.1371/journal.pone.0236069)

**S2 Fig.** (A, B) Oil Red O staining by duck adipocytes transfected with si-circ-PLXNA1 and miR-214 mimics for 48 h and differentiation for 3 d. (C, D) Oil Red O staining by duck adipocytes transfected with si-CTNNB1 and miRNA-NC for 48 h and differentiation for 3 d. (E, F) Oil Red O staining by duck adipocytes transfected with si-NC or miRNA-NC for 48 h and differentiation for 3 d.


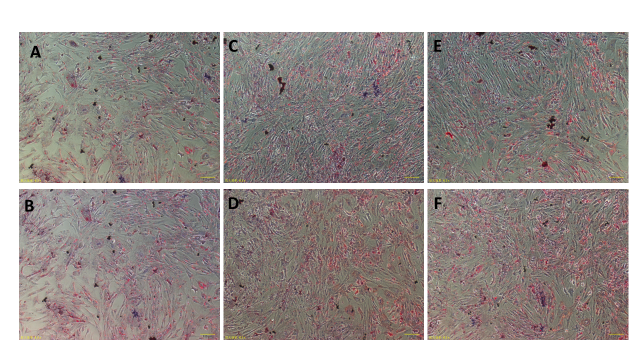

Supplement: S2 Fig — (A, B) Oil Red O staining by duck adipocytes transfected with si-circ-PLXNA1 and miR-214 mimics for 48 h and differentiation for 3 d. (C, D) Oil Red O staining by duck adipocytes transfected with si-CTNNB1 and miRNA-NC for 48 h and differentiation for 3 d. (E, F) Oil Red O staining by duck adipocytes transfected with si-NC or miRNA-NC for 48 h and differentiation for 3 d. (DOCX) [file pone.0236069.s002.docx]
