# Supplementary material for: Circular RNA expression profiling reveals that circ-PLXNA1 functions in duck adipocyte differentiation
Source: PLoS One. 2020 Jul 21;15(7):e0236069. doi: 10.1371/journal.pone.0236069 (PMC7373283; doi:10.1371/journal.pone.0236069)
Supplement: S1 Raw images — (PDF) [file pone.0236069.s003.pdf]

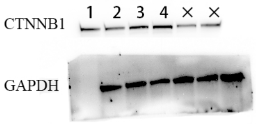

1,2 : CVC

3,4: CVT

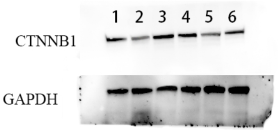

1,2: si-circ-PLXNA1+miR-214 mimics

3,4: si-NC+miRNA-NC

5,6: si-CTNNB1+miRNA-NC

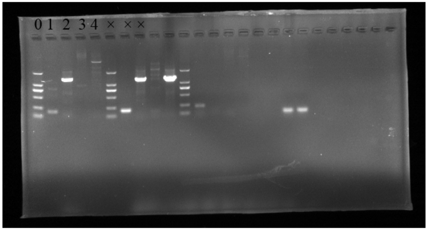

0: 2000bp marker

1: cDNA divergent primer

2: cDNA convergent primer

3: gDNA divergent primer

4: gDNA convergent primer
