# Supplementary material for: Circular RNA expression profiling reveals that circ-PLXNA1 functions in duck adipocyte differentiation
Source: PLoS One. 2020 Jul 21;15(7):e0236069. doi: 10.1371/journal.pone.0236069 (PMC7373283; doi:10.1371/journal.pone.0236069)
Supplement: S1 Table — (DOCX) [file pone.0236069.s004.docx]

**S1 Table.**  Overview of sequencing data

| Sample | Raw Reads | Clean Reads | Clean Bases |
| --- | --- | --- | --- |
| CVC1 | 120,595,196 | 115,755,900 | 17.36G |
| CVC2 | 96,371,798 | 91,946,634 | 13.79G |
| CVC3 | 97,452,648 | 93,339,178 | 14G |
| CVT1 | 88,452,074 | 84,430,582 | 12.66G |
| CVT2 | 98,659,728 | 94,315,726 | 14.15G |
| CVT3 | 101,660,678 | 96,920,942 | 14.54G |
| Average | 100,532,020 | 96,118,160 | 14.42G |
